# Host range, host specificity and hypothesized host shift events among viruses of lower vertebrates

**DOI:** 10.1186/1297-9716-42-67

**Published:** 2011-05-18

**Authors:** Isabel Bandín, Carlos P Dopazo

**Affiliations:** 1 Unidad de Ictiopatología-Patología Viral, Departamento de Microbiología y Parasitología, Instituto de Acuicultura, Universidad de Santiago de Compostela, Spain

## Abstract

The successful replication of a viral agent in a host is a complex process that often leads to a species specificity of the virus and can make interspecies transmission difficult. Despite this difficulty, natural host switch seems to have been frequent among viruses of lower vertebrates, especially fish viruses, since there are several viruses known to be able to infect a wide range of species. In the present review we will focus on well documented reports of broad host range, variations in host specificity, and host shift events hypothesized for viruses within the genera *Ranavirus*, *Novirhabdovirus*, *Betanodavirus*, *Isavirus*, and some herpesvirus.

## 1. Introduction

The successful replication of a viral agent in a host is a complex process which consists of a number of interactions, most of them related to the coevolution of pathogen and host. This coevolution often leads to a species specificity of the virus and can make interspecies transmission difficult. Therefore, natural host range switches by viruses are rare events. However, when they occur the results can become severe because the viruses may then spread widely through non previously adapted, and therefore immunologically naïve host populations.

Upon transmission to a new host species, viruses must usually adapt to a new genetic and immunologic environment in order to replicate and spread to other individuals within the species [1]. The high rates of mutation and replication of RNA viruses, such as human immunodeficiency virus (HIV) and influenza, facilitate the occurrence and fixation of those mutations that become beneficial under certain conditions [2]. Viral adaptations to new hosts primarily manifest as amino acid substitutions which can allow more efficient virus cell entry into the new host [3,4], block interactions with detrimental host proteins [5,6] or promote escape from both the new and the old host's immune responses [7,8].

Influenza A is the paradigm of a virus capable of interspecies and interclass transmission. Those viruses are found in humans as well as in other animals, including swine, horses and birds, waterfowl being considered the natural reservoir [9]. Subtypes of Influenza A are distinguished by the two surface glycoproteins: haemagglutinin (HA) and neuraminidase (NA). Periodically a subtype of influenza can make the shift from aquatic birds to humans, possibly through an intermediate host, resulting in a widespread pandemic in an immunologically naïve population. These antigenic shifts can occur either through the transfer of an entire virus from one host to another or through a reassortment process where genomic segments of the avian virus mix with genomic segments of a virus currently circulating in humans.

A number of proteins have been implicated in determining host specificity of the virus. Influenza haemagglutinin binds to sialic acid linked to galactose on the surface of the targeted cell, and the differing nature of the sialic acid-galactose linkages in birds and humans provides an important barrier to host shift events. In this sense, a number of amino acid substitutions have been produced in influenza haemagglutinin to adjust to the different receptors [10-14]. Neuraminidase, the protein responsible for cleaving the haemagglutinin from the receptor surface, also seems to be adapted to the particular sialic acid linkages [15]. Proteins in the viral replication complex (PA, PB1, PB2 and NP) have also been implicated in limiting host range by restricting replication and intra-host spread in mammals (for a review see [16]). In particular, a specific substitution in the PB2 gene has been identified as crucial for replication and intra-host spread in mammals [17-19].

Severe acute respiratory syndrome coronavirus (SARS-CoV) is a recently identified human coronavirus. The extremely high homology of the viral genomic sequences between the viruses isolated from humans (huSARS-CoV) and those of palm civet origin (pcSARS-CoV) suggested possible palm civet-to-human transmission. Genetic analysis revealed that the spike (S) protein of pcSARS-CoV and huSARS-CoV was subjected to the strongest positive selection pressure during transmission, and there were six amino acid residues within the receptor binding domain of the S protein that were potentially important for SARS progression and tropism. It has been demonstrated that the double substitution of two amino acid residues of pcSARS-CoV for those of huSARS-CoV made pcSARS-CoV capable of infecting human cells [4], suggesting that these two residues are involved in the palm civet-human transmission.

Under certain circumstances, even a genetically stable DNA virus can gain the mutation required to adapt to a new host. That is the case of canine parvovirus (CPV) which emerged in 1978 as the cause of new enteric and myocardial diseases in dogs. The new virus spread globally in a pandemic and has since remained endemic in dogs throughout the world [20,21]. Phylogenetic analysis showed that all CPV isolates obtained so far, termed CPV type 2, descended from a single ancestor closely related to the feline panleukopenia virus (FPV) which infects cats, mink and raccoons, but not dogs or cultured dog cells [21]. FPV and CPV type 2 isolates differ by as little as 0.5% in DNA sequence and it is possible that changes of only two amino acid residues in the capsid protein could have introduced the canine host range [22,23]. During 1979 a CPV variant (CPV type 2a) emerged, spread worldwide within 1 year and replaced the CPV type 2 strain. CPV type 2a contained five substitutions in the capsid sequence compared to CPV type 2 and also infected and caused disease in cats [24-26]. Therefore, the emergence of CPV seems to have been a multistep process, where a small number of mutations in the capsid protein gene allowed the virus to efficiently infect and spread within a new host order [27].

Viruses of lower vertebrates include a large number of viral agents, belonging to different viral families and genera, with RNA and DNA genomes, and displaying different host specificities. In fact, some viruses have a very narrow host range, whereas others are known to be able to infect a wide range of species. The wide host range suggests that, in any moment along the viral evolution, those viruses may have been involved in different host shift events. In the present review we will focus on well documented or hypothesized cases of host shift as well as variations in host range for the genera *Ranavirus*, *Novirhabdovirus*, *Betanodavirus*, *Isavirus *and several herpesvirus. However, the suspicion for interspecies transmission in other fish viruses remains.

## 2. Ranaviruses - Interspecies and interclass transmission

Iridoviruses are large double stranded DNA viruses with an icosahedral capsid ranging from 120 to 350 nm in diameter. Ranavirus is one of the five genera within the family *Iridoviridae*. Among the five genera, two contain viruses of invertebrates (genera *Chloridiridovirus *and *Iridovirus*), whereas the remaining three genera *(Lymphocystivirus*, *Megalocytivirus *and *Ranavirus*) contain viruses that infect lower vertebrates [28]. The family also includes several viruses than remain unassigned to any genus. None of the iridoviruses are known to infect homeothermic vertebrates. A variety of molecular characteristic such as GC content, nucleotide sequence and inferred amino acid sequence of key genes, such as the major capsid protein gene (MCP), can be used to distinguish genera and species within genera [29].

Although the disease was known before [30], the lymphocystis disease virus (LCDV), the first known iridovirus was discovered in 1962 [31]. Since then, iridoviruses have been linked to disease in frogs, salamanders and other amphibians, reptiles [29,32], and more than 140 wild and cultured fish species in different parts of the world [29,32,33]. Interestingly, most of these fish iridoviruses have been shown to be more closely related to frog virus 3, the type species of the genus *Ranavirus*, than to *Lymphocystivirus*. In fact, ranaviruses have become important pathogens for cultured and wild finfish, not only due to the severity of the diseases that they cause but also because of their rapid global emergence in recent years [34].

Members of the genus *Ranavirus *infect vertebrates of three different taxonomic classes: amphibians, reptiles and fish [35]. Since the identification of epizootic hematopoietic necrosis virus (EHNV), first isolated from redfin perch [36] and the first iridovirus associated with epizootic mortality in vertebrates [37], ranaviruses have caused epizooties in other fish species: sheatfish and catfish in Europe [38], largemouth bass [39] and ornamental fish (imported from Asia) in the USA [40], as well as grouper cultured in Asia [41,42]. In addition, ranaviruses have been isolated from diseased frogs, salamanders, turtles and snakes in different parts of the world [43-47]. At present the International Committee on Taxonomy of Viruses [48] recognizes six species within the genus based on analysis of host range, sequence identity, and protein and RFLP profiles [49,50], but there are also many additional isolates as well as a number of tentative species (Table 1). FV-3, EHNV, *Bhole Iridovirus *(BIV), *Ambystoma tigrinum virus *(ATV) and *European catfish virus *(ECV) are five closely-related viral species that share over 90% sequence identity within the MCP and other genes, but clearly differ from each other in host range and RFLP profiles. The sixth species is Santee-Cooper ranavirus (SCRV) that along with Singapore grouper iridovirus (SGIV), a tentative species, are the most divergent members of the genus. The MCP genes of these two viruses show approximately 80% and 70% sequence identity, respectively, with the MCP genes of the 5 other ranavirus species [51,52].

**Table 1:** Ranaviruses recognised by the ICTV.

**Virus species**^**1 **^**or isolates**	Host species	Geographic range
*Ambystoma tigrinum virus *(ATV)	Tiger salamander; South American frog (95% similarity)^2^	North Dakota, Utah, USA; Northern Patagonia, Argentina
Regina ranavirus	Tiger salamander; South American frog (95% similarity)	Southern Canada; Arizona, USA, Northern Patagonia, Argentina
*Bohle iridovirus *(BIV)	Burrowing frog; tilapia (*Oreochromis mossambicus*) Australian anurans (experimentally); Barramundi (experimentally). Giant toad (sero-related)	Northern and Northeastern Australia. Venezuela
*Epizootic haematopoietic necrosis virus *(EHNV) EHNV-related	Redfin perch; rainbow trout	Australia
	Pikeperch	Denmark Finland
*European catfish iridovirus *(ECV)	Catfish; Black bullhead (experimentally)	France, Italy
European sheatfish iridovirus (ESV)	Sheatfish	Germany
*Frog virus *3 (FV-3)	Giant toad (sero-related);	Venezuela
	Tiger frog	Thailand
	Hermann's tortoise	Switzerland
	Pig frog	China
	Spotted salamander	Southern Ontario, Canada
	Green frog, American bullfrog	Tennessee, USA; Brazil
Box turtle virus 3	Box turtle	USA
Bufo bufo United Kingdom virus	Common toad	UK
Lucké triturus virus 1	Frog	USC
Rana temporaria United Kingdom virus Bufo marinus Venezuelan iridovirus 1	Eur. common frog Giant toad	UK Venezuela and Australia
Redwood Park virus	Red-legged frog tadpole	USA
Stickleback virus	Threespine stickleback	USA
Tadpole virus 2 and Tadpole edema virus	Common frog, Green frog, red-leg frog	France, North America
Tiger frog virus (TFV)	Tiger frog	Thailand, China
Tortoise virus 5	Tortoise	USA
*Santee Cooper ranavirus *(SRCV),	Largemouth bass; black crappie	USA
Doctor fish virus (DFV)	Doctor fish	North America (first imported from) Asia
Guppy virus 6 (GV6)	Guppy	North America (first imported from) Asia
Largemouth bass virus (LMBV)	Largemouth bass	USA
		
	Edible frog	Italy
	Grouper	Singapore
	Hermann's tortoise	Switzerland

1. The five ranavirus species recognised by the ICTV are shown in italics.

2. The virus isolated from frog showed 95% sequence similarity with the type species.

From reviews by Holopainen et al. [67], Mao et al. [50], Whittinton et al. [32] and Williams et al. [29].

**Fish**: tilapia (*Oreochromis mossambicus*); barramundi (*Lates calcarifer*); redfin perch (*Perca fluviatilis*); rainbow trout (*Oncorhynchus mykiss*); turbot (*Scophthalmus maximus); *pikeperch (*Stizostedion lucioperca*); Catfish (*Ictalurus melas*); black bullhead (*Ameiurus melas*); sheatfish (*Silurus glanis*); threespine stickleback (*Gasterostelus aculeatus*); Largemouth bass (Micropterus salmoides); black crappie (Pomoxis nigromaculatus); doctor fish (Labroides dimidatus); guppy (Poecilia reticulata); grouper (Epinephelus tauvina). **Amphibians**: Tiger salamander (*Ambystoma tigrinum*); South American frog (*Atelognathus patagonicus*); burrowing frog (Limnodynastes ornatus); Australian anurans (*Litorea terraereginae *and *L. latopalmata*); giant toad (*Bufo marinus*); tiger frog (*Rana tigrina*); pig frog (*Rana grylio*); spotted salamander (*Ambystoma maculatum*); green frog (*Rana clamitans*), American bullfrog (*R. catesbeiana*); common toad (*Bufo bufo*); pipiens frog (*Rana pipiens*); European common frog (*Rana temporaria*); red-legged frog (*Rana aurora*); common frog (*Rana temporaria*); edible frog (Pelophylax esculentus). **Reptiles**: Hermann's tortoise (*Testudo hermanni*); box turtle (*Terrapene carolina carolina *and *T. carolina bauri*); tortoise (*Testudo horsfieldi*).

Several of these viruses have been demonstrated to have broad host specificity, suggesting the potential for interspecies and interclass transmission. A good example is BIV, which was isolated originally from diseased ornate burrowing frog (*Limnodynastes ornatus*) tadpoles in Australia [53] and has been shown experimentally to be pathogenic for other species of frog [54,55], and also for a fish species, barramundi (*Lates calcarifer*) [56,57]. Moreover, BIV has been associated with the "spinning tilapia" syndrome which causes epizootic mortalities in fry populations of tilapia (*Oreochromis mossambicus*) [56,58]. A case of interspecies transmission was demonstrated by Cunningham et al. [59], who infected common frogs *Rana temporaria *with two ranavirus isolates obtained from diseased toads (*Bufo bufo*). In addition, apparently identical FV3 strains were isolated from dead or moribund free-living threespine stickleback fish (*Gasterosteus aculeatus*) and sympatric tadpoles of the red-legged frog (*Rana aurora*) [60], and FV3 and FV3-like viruses have been reported to infect sympatric amphibian species including ranid and hylid tadpoles, larval salamanders and newts [61]. Moreover, it has been experimentally demonstrated that FV3·is pathogenic for pike (*Esox lucius*) [62]. A very interesting case of cross-class infection was found studying ATV infection of salamanders. Phylogenetic analyses of sequence data from the MCP gene of ATV isolates from very different locations indicate that they are more closely related to fish ranaviruses, such as EHNV, than to other amphibian ranaviruses, such as FV3 [63]. These data suggested that ATV possibly originated via a host switch from fish, and was spread across North America due to the substantial trade of salamander larvae sold for bait [63-65].

In a very recent and interesting study Jancovich et al. [66] obtained evidence for host shifts among ranaviruses and proposed that the ancestral ranavirus was a fish virus. Those authors performed a dot plot comparison of the EHNV genome with that of other ranaviruses previously sequenced (ATV, FV, TFV, Grouper iridovirus and SGIV) and the results obtained indicated that EHNV is more closely related to the amphibian ranaviruses than to the GIV-like viruses infecting fish, as shown by other phylogenetic analyses previously performed [67]. In fact, Jancovich et al. [66] observed two lineages, FV3/TFV (frog lineage) and EHNV/ATV (fish/salamander lineage), and the existence of two major genomic inversions that can be visualised on the dot plot. These inversions would correspond to rearrangements of segments in the FV3-like lineage, which means that EHNV/ATV is closer to the most recent common ancestor (MRCA) of ranaviruses. These authors postulate that there must have been at least three species jumps, from fish to frogs, from fish to salamanders and from frogs to reptiles, and perhaps as many as four species jumps, including a jump from tetrapod amphibians back to fish. A new ranavirus isolate obtained recently from dead wild edible frogs (*Pelophylax esculentus*) in Denmark, which showed a 98.8% nucleotide identity in the MCP gene with EHNV [68], would support that hypothesis.

It has been suggested that after the divergence into the salamander virus and frog virus lineages a subsequent host specific evolution could have occurred that would have limited cross transmission between both hosts, at least in laboratory infections [47]. However, there are some data that indicate that ranavirus transmission between these species occurs in nature. In this sense, an FV-3-like virus has been isolated from spotted salamander suffering from mortalities [69], and a model of FV3/FV3-like virus transmission in aquatic amphibian communities postulates that transmission of the virus occurs between anuran (i.e. frogs) and urodele (i.e. salamanders) species [61]. There is also some evidence that salamander ranavirus isolates are also isolated from or detected in laboratory-infected frogs [70].

## 3. Betanodaviruses - The role of mutation and reassortment in host specificity

Piscine nodaviruses belong to the genus *Betanodavirus*, within the family *Nodaviridae *[71]. Betanodaviruses are the aetiological agents of the disease known as viral nervous necrosis (VNN) or viral encephalopathy and retinopathy (VER), a devastating neuropathological condition that affects marine fish worldwide [72]. The affected fish developing clinical signs show abnormal swimming, neurological problems and buoyancy control loss.

The disease typically occurs in an outbreak form in larval and juvenile fish, and several species have been shown to be specially affected such as sea bass (*Lates calcarifer *and *Dicentrarchus labrax*), groupers (*Epinephelus akaara, Epinephelus fuscogutatus, Epinephelus malabaricus, Epinephelus moara, Epinephelus septemfasciatus, Epinephelus tauvina, Epinephelus coioides *and *Cromileptes altivelis*), striped jack (*Pseudocaranx dentex*), parrotfish (*Oplegnathus fasciatus*), tiger puffer (*Takifugu rubripes*), and flatfish (*Verasper moseri*, *Hippoglossus hippoglossus*, *Paralichthys olivaceus*, *Scophthalmus maximus*) [73]. The affected fish species and geographical ranges of clinical VNN described so far are provided in Table 2.

**Table 2:** Fish species affected -in natural infections- by viral nervous necrosis (VNN).

Family	Common name	Species	References	Geografic Range
*Anguillidae*	European eel	*Anguilla anguilla*	[146,147]	Taiwan
*Carangidae*	Striped Jack	*Pseudocaranx dentex*	[148]	Japan
	Purplish amberjack	*Seriola dumerili*	[149]	Japan
	Pompano	*Trachinotus blochii*	[146,147]	Taiwan
		*T. falcatus*	[146,147]	Taiwan
*Centropomatidae*	Barramundi	*Lates calcarifer*	[73,146,147,150-157]	Taiwan, India, Singapore, Malaysia, Australia, Israel, Tahiti, Indonesia
	Japanese sea bass	*Lateolabrax japonicus*	[158]	Japan
*Cichlidae*	Tilapia	*Oreochromis niloticus*	[159]	Europe
*Eleotridae*	Sleepy cod	*Oxyeleotris lineolatus*	[73]	Australia
*Gadidade*	Atlantic cod	*Gadus morhua*	[160-163]	Atlantic Canada, Atlantic USA, Norway, UK
	Haddock	*Melanogrammus aeglefinus*	[160,161]	Atlantic Canada, Atlantic USA
*Percichthydae*	Sea bass	*Dicentrarchus labrax*	[156,164-168]	Martinique, Italy, Greece, Spain, Malta, Portugal, Israel
*Serranidae*	White grouper	*Epinephelus aeneus*	[156]	Israel, Philippines
	Red spotted grouper	*E akaara*	[169, 170,	Taiwan, Japan
	Yellow grouper	*E. awooara*	146, 147, 171]	Taiwan
	Orange-spotted grouper	*E. coioides*	[172]	Philippines
	Blackspotted grouper	*E. fuscogutatus*	[171]	Taiwan
	Brownspotted grouper	*E. malabaricus*	[168,173]	Thailand
	Dusky grouper	*E. marginatus*	[73]	Mediterranean
	Kelp grouper	*E. moara*	[174]	Japan
	Sevenband grouper	*E. septemfasciatus*	[175,176]	Japan, Korea
	Greasy grouper	*E. tauvina*	[73,177]	Malaysia, Phillipines, Singapore
	Humpback grouper	*Chromileptes altivelis*	[146,147,178]	Taiwan, Indonesia
	Spottet coral grouper	*Plectropomus maculatus*	[179]	Thailand
*Latridae*	Striped trumpeteer	*Latris lineata*	[73]	Australia
*Lutjanidae*	Firespot snapper	*Lutjanus erythropterus*	[146,147]	Taiwan
*Monacanthidae*	Thread-sail filefish	*Stephanolepis cirrhifer*	[180]	Thailand
*Mugilidae*	Striped mullet	*Mugil cephalus*	[156]	Israel
	Golden mullet	*Liza auratus*	[181]	Caspian sea (Iran)
*Oplegnathidae*	Japanese parrotfish	*Oplegnathus fasciatus*	[182]	Japan
	Rock porgy	*O. punctatus*	[148,168]	Japan
*Paralicthyidae*	Japanese flounder	*Paralichthys olivaceus*	[183]	Japan
*Pleuronectidae*	Barfin flounder	*Verasper moseri*	[149]	Japan
	Halibut	*Hippoglossus hippoglossus*	[184,185]	Norway, UK
	Winter flounder	*Pleuronectes americanus*	[160]	Atlantic Canada
*Plotosidae*	Catfish	*Tandanus tandanus*	[73]	Australia
*Poecilidae*	Guppy	*Poecilia reticulata*	[186]	Singapore
*Rachycentridae*	Cobia	*Rachycentron canadum*	[146]	Taiwan
*Sciaenidae*	Red drum	*Sciaenops ocellatus*	[156,187]	Korea, Israel
	Shi drum	*Umbrina cirrosa*	[168,188,189]	France, Italy
	White seabass	*Atractoscion nobilis*	[190]	California (USA)
*Scophthalmidae*	Turbot	*Scophthalmus maximus*	[191]	Norway
*Sebastidae*		*Sebastes oblongus*	[192]	Korea
*Siluridae*	Chinese catfish	*Parasilurus asotus*	[147]	Taiwan
*Soleideae*	Dover sole	*Solea solea*	[163]	UK
	Senegalese sole	*Solea senegalensis*	[82]	Iberian Peninsula
*Sparidae*	Gilthead sea bream	*Sparus aurata*	[82,193-195]	Israel, France, Italy, Iberian Peninsula
*Triodontidae*	Tiger puffer	*Takifugu rubripes*	[174]	Japan

Betanodaviruses are small (25-30 nm), nonenveloped, icosahedral RNA viruses. The genome consists of two single stranded, positive-sense molecules. The larger genomic segment, RNA1 (3.1 kb), encodes the RNA dependent RNA polymerase (RdRp) of approximately 100 kDa, also named protein A [74,75]. The smaller segment, RNA2 (1.4 kb), encodes the capsid protein of about 42 kDa [74,76]. In addition, a subgenomic RNA3 is synthesised during RNA replication from the 3' terminus of RNA1.

Betanodaviruses have been classified into four genotypes, designated SJNNV (striped jack nervous necrosis virus), TPNNV (tiger puffer nervous necrosis virus), RGNNV (red grouper nervous necrosis virus) and BFNNV (barfin flounder nervous necrosis virus), using a partial sequence of RNA2, the T4 region, which is a highly variable region of around 400 nt [77,78]. These types exhibit different capabilities for infecting fish species. Thus, RGNNV shows the broadest host range and causes disease in a variety of warm-water fish species, BFNNV is restricted to cold-water marine fish species and TPNNV infects only one species [72]. With regards to the SJNNV type, although for several years it was considered to be restricted to a few species present in Japanese waters [72,78], in recent years it has been found in Senegalese sole *Solea senegalensis *[79,80] as well as gilthead sea bream *Sparus aurata *and sea bass cultured in the Iberian Peninsula [79]. More recent studies [81] reported that most of the betanodavirus strains infecting Senegalese sole and gilthead sea bream, previously typed as SJNNV on the basis of the T4 region, were in fact RGNNV/SJNNV reassortants. Olveira et al. [81] observed that the reassortant strains exhibited a slightly modified SJNNV capsid, with three different amino acid positions in all strains (the differences increased to a maximum of six in some strains). One of these changes observed in residue 247 was encoded by the nucleotide triplet 737-739, which was included in the region between nucleotides 695 and 765, described previously by Ito et al. [82] as a host specificity determinant. Another change in the amino acid sequence at residue 270 was also observed on the C-terminal side of the capsid protein. These results confirmed that C-terminal protruding domains of the capsid protein are involved in host specificity, as reported previously by Iwamoto et al. [83] and Ito et al. [82]. It is well known that even a small number of amino acid substitutions in the capsid proteins can have dramatic effects on the host specificity of different animal viruses [84]. In this case, the changes observed in the SJNNV capsid seem to have allowed it to efficiently infect and spread within two new hosts, causing epizootic outbreaks in Senegalese sole and gilthead sea bream, which were not previously considered susceptible to SJNNV.

Other authors have also reported the existence of reassortants among betanodavirus isolates obtained from symptomatic sea bass harbouring an RNA1 segment of SJNNV type and an RNA2 of RGNNV type [85]. These data indicated that both combinations of genomic segments of SJNNV and RGNNV genotypes are successful and allow the resultant reassortant strains to produce disease. Interestingly, a certain relationship between the type of reassortant and the susceptible host species seems to exist: SJ/RG affecting sea bass and RG/SJ affecting Senegalese sole and gilthead sea bream.

Souto et al. [86] experimentally demonstrated the pathogenicity of the reassortant RG/SJ strains for Senegalese sole and compared it to that of the parental strains (RGNNV and SJNNV). Mortality was recorded only in the fish infected with the RG/SJ strains and betanodavirus were re-isolated from dead fish, fulfilling the River's postulates. However, virus was detected by RT-PCR and isolated from all pools of fish inoculated with RGNNV and SJNNV strains. These results indicate that both genotypes can replicate in Senegalese sole with no evident pathological effects and that the changes produced after the reassortment account for the pathogenicity for Senegalese sole.

## 4. Novirhabdoviruses - Infectious haematopoietic necrosis virus and viral haemorrhagic septicaemia virus, two different strategies within the same genus

*Novirhabdovirus *is one of the six established genera within the family *Rhabdoviridae*, and it is one of the two genera of this family known to infect aquatic animals (along with the *Vesiculovirus *genus). Two of the four recognised species of the genus are *infectious haematopoietic necrosis virus *(IHNV), the aetiological agent of infectious haematopoietic necrosis (IHN), and *viral haemorrhagic septicaemia virus *(VHSV), the causative agent of viral haemorrhagic septicaemia (VHS). Novirhabdovirus posses**s **enveloped bullet-shaped virions. The viral genome consists of a linear non-segmented, negative-sense, single-stranded RNA of approximately 11 kilobases and contains 6 genes in the order 3'-N-P-M-G-NV-L-5' [87]. These viral species are quite different in terms of host range: quite narrow in the case of IHNV -apparently limited to salmonid fish-, and very broad for VHSV, including diverse fresh water and marine fish species.

IHNV is the type species for the genus *Novirhabdovirus *and it is one of the most serious viral pathogens of salmonid fish, infecting wild [88] and cultured salmonids in the USA, Europe and Asia [89]. The virus causes an acute systemic disease that can affect all five species of Pacific salmon (sockeye salmon *Oncorhynchus nerka*, pink salmon *O. gurbuscha*, chinook salmon *O. tshawytscha*, chum salmon *O. keta*, and coho salmon *O. kisutch*,) as well as Atlantic salmon (*Salmo salar*), and rainbow trout (*O. mykiss*) [89]. However, not all salmonid species are equally susceptible to IHNV [90-93]. Garver et al. [90] have reported differences in susceptibility of sockeye salmon and rainbow trout to the different phylogenetic groups of IHNV established in North America (U, M and L) [94]. Isolates belonging to the U genogroup were highly virulent for sockeye salmon, while the M genogroup IHNV isolates were highly virulent for rainbow trout. Although not demonstrated, the U genogroup specificity for sockeye salmon is hypothesised to be associated with long-term coevolution of IHNV with sockeye salmon over centuries [90,94]. In contrast to the U genogroup situation, the M genogroup specificity for rainbow trout may reflect a relatively recent host-parasite interaction. The origin of the M genogroup may have involved a host shift of the U genogroup IHNV from sockeye salmon to rainbow trout during the 1970s, followed by a relatively rapid evolution and divergence in rainbow trout [94,95]. If this hypothesis is true, it would be interesting to know the mechanisms involved in the adaptation of the virus to the new host as this apparently caused a loss of virulence for its original host.

Until the mid-1980s, VHS was regarded as a disease affecting only rainbow trout and a few other freshwater fish species in aquaculture in continental Europe. Since then, however, VHSV has been isolated from a large range of free-living marine fish species, either diseased or asymptomatic, throughout the northern hemisphere. So far, VHSV has been isolated from more than 70 different fish species (for a review see references [96,97]).

Different studies based on different gene sequences, including nucleoprotein (N), glycoprotein (N) and non-structural (NV) protein genes, have identified the existence of four genotypes of VHSV [98-101]. Genotype I group isolates from continental Europe are pathogenic for rainbow trout, as well as several marine isolates from the Baltic sea; genotype II includes a number of marine isolates obtained from the Baltic sea with no clear link to rainbow trout aquaculture; genotype III comprises isolates from around the United Kingdom and the Flemish Cap area in the Northwestern Atlantic Ocean, and genotype IV includes VHSV strains isolated from Korea and Japan, both the Pacific and the Eastern coast of North America, and more recently the Great Lakes region.

The use of phylogenetic tools has provided considerable genetic evidence indicating that rainbow trout pathogenic VHSV emerged from a genotype I-type marine ancestor [98,101-103]. The shift could be explained by the occurrence of a single introduction or adaptation event followed by expansion of this "new" genotype virus within trout aquaculture [98,101]. It has been suggested that the feeding of unpasteurised raw marine fish to farmed fish, a common practice in the early days of fish farming, could have been a likely route for the introduction of marine VHSV within rainbow trout aquaculture [104]. Only a limited number of amino acid residues might be involved in the determination of VHSV virulence for salmonids and this highlights the potential risk that marine strains may pose to freshwater aquaculture [105]. Snow & Cunningham [106] observed an increase in the virulence of the turbot isolate 860/94 following a number of passages in rainbow trout, although that increasing virulence was not accompanied by a difference in the consensus sequence in the glycoprotein.

Some phylogenetic studies indicate that VHSV may have been present in marine fish species in Europe for centuries and that the genotypes became separated a long time before fish farming was established in Europe and North America [107]. However, no isolates from wild marine fish were included in this study. Subsequent studies on molecular clocks supported this hypothesis and showed the existence of a molecular clock for European marine isolates without positive selection and a molecular clock for European freshwater isolates with positive selection [98]. In this sense, it has been estimated that the North American and European VHSV types diverged around the year 1500, and that the European freshwater and marine isolates diverged around 1950 [98].

In spite of the lack of reports on the definition of molecular determinants involved in host specificity, some studies performing comparative analysis of the complete genome sequences provide clues to the possible involvement of a small number of nucleotides [105,108]. To demonstrate their implication in host specificity, the availability of infectious clones to generate recombinant IHNV and VHSV viruses will be helpful (see Biacchesi [109]).

## 5. Infectious salmon anaemia virus: Orthomyxoviruses "made for the change"

Infectious salmon anaemia virus (ISAV), the etiological agent of infectious salmon anaemia (ISA), is an RNA virus of the family *Orthomyxoviridae*, the only member of the genus *Isavirus *[110]. The genome of ISAV consists of eight segments of linear negative-sense single- stranded RNA. Viral particles are enveloped, with a diameter of 90-140 nm, and show surface projections consisting of a combined haemagglutinin-esterase (HE) protein encoded on segment 6 [111] and a separate fusion (F) protein encoded on segment 5 [112].

ISA is characterized by high mortality, and natural outbreaks have only been described in farmed Atlantic salmon. However, ISAV has been reported in both wild salmonid and non-salmonid fish [113-115], and the virus may, under experimental conditions, persist and replicate in other salmonid (*Salvelinus alpinus, O. mykiss, O. keta, O. kisutch*) [116-119] and non-salmonid fish (*Clupea harengus, Gadus morhua*) [120,121].

The HE surface glycoprotein is the molecule with the highest sequence variability, and is assumed to be of importance in determining virulence. Most of the variation in this molecule is concentrated on a small highly polymorphic region (HPR). It is widely assumed that the source of the virulent ISAV isolates is an ISAV variant- designated HPR0- without any deletion in the HPR gene. The non-virulent nature of HPR0 viruses was indicated by the lack of disease in vivo and by their failure to replicate in cell culture [113,122]. The widely held model suggests that virulent variants of the HPR0 archetype arise by deletion of several nucleotides in the HPR [113,123,124]. The driving forces behind the differential deletion patterns in the HPR could be analogous to a phenomenon described for Influenza A neuraminidase, where varying lengths of the stalk region have been reported, a property that was associated to host range adaptation [125,126]. Following this theory and on the basis of a phylogenetic analysis of the HPR region, Mjaaland et al. [123] suggested that European ISA outbreaks may have been the result of several independent introductions of virus into farmed Atlantic salmon from wild fish, followed by adaptation to the new host through parallel but varied hemagglutinin gene deletions.

A recent study by Markussen et al. [127] has provided evidence for the role of recombination and reassortment in the evolution of ISAV. Those authors have demonstrated the existence of a new marker of virulence next to one of the two potential cleavage sites in the F protein and suggest that a single amino acid mutation may alter the recognition site, having a direct effect on the virulence of the virus. Markussen et al. [127] also suggested that the alterations at the cleavage site of the ISAV F protein together with deletions in the HPR region, most likely represent an adaptation of ISAV to Atlantic salmon from an unidentified reservoir, which leads to disease in densely populated fish farms.

However, Kibenge et al. [128] have postulated an alternative evolutionary model, which, in contrast to the widely accepted deletion theory, suggests that the original ancestral ISAV was virulent and that the insertion of specific motifs resulted in its attenuation. This last theory would not support a wild origin of ISAV because "wild" viruses are expected not to be as virulent as the farming-associated viruses. In natural conditions a balance between the virus and the host is expected to be maintained. However, this balance will be broken under intensive rearing conditions, conducive to an increase of virulence.

## 6. Herpesviruses: Very host specific viruses?

Herpesviruses (HVs) infect a wide variety of vertebrate hosts including mammals, birds, reptiles, amphibians and fish, and at least one invertebrate group, bivalve molluscs. HV share a characteristic virion structure, which consist**s **of a large, linear, double-stranded DNA genome, an icosahedral capsid, a proteinaceous matrix (the tegument) and an envelope containing viral proteins [129].

HV taxonomy has recently undergone a revision by the ICTV [48], in which the previous family *Herpesviridae *was raised to the order *Herpesvirales *and split into three families: *Herpesviridae*, which divides into the subfamilies *Alpha, Beta *and *Gammaherpesvirinae*, containing mammalian, avian and reptilian viruses; *Alloherpesviridae *containing fish and amphibian viruses; and *Malacoherpesviridae *containing one single virus *Ostreid herpesvirus *(OsHV-1). Table 3 presents a list of fish and amphibian HV isolated in cell culture.

**Table 3:** Members of the family *Alloherpesviridae *and other fish herpesvirus isolated in cell culture.

Genus	Viral species	Common name (abbreviation)
*Cyprinivirus*	*Cyprinid herpesvirus 1*	
	*Cyprinid herpesvirus 2*	
	*Cyprinid herpesvirus 3*	Koi Herpesvirus (KHV)
*Ictalurivirus*	*Ictalurid herpesvirus 1*	Channel cat fish virus (CCV)
	*Ictalurid herpesvirus 2*	Ictalurus melas herpesvirus (ICmHV)
	*Acipenserid herpesvirus 2*	White sturgeon HV2
*Salmonivirus*	*Salmonid herpesvirus 1*	HV salmonis (HPV)
	*Salmonid herpesvirus 2*	Oncorhynchus masou virus (OMV)
		Yamame tumor virus (YTV)
		Oncorhynchus kisutch virus (OKV)
		Coho salmon tumor virus (COTV)
		Coho salmon herpes virus (CSH)
*Batrachovirus*	*Ranid herpesvirus 1*	Lucké tumor HV (LTHV)
	*Ranid herpesvirus 2*	Frog virus 4 (FV-4)
Other herpesvirus		
	Anguillid herpesvirus 1	HV anguillae
	Percid herpesvirus 1	HV vitreum, walleye HV
		

As a general rule, the natural host range of mammalian and avian HV is highly restricted, and most herpesviruses are thought to have evolved in association with single host species [129], but exceptions have been reported among mammals [130]. On the basis of a comparative phylogenetic study of different hosts and fish herpesviruses, Waltzek et al. [131] indicate that some fish (salmonid, ictalurid and ciprinid) and ranid HV may have coevolved with their hosts, at least at the tips of the phylogenetic tree. However, the phylogenetic analysis revealed an overall discordance between HV and host lineages. One example of these discordances is provided by the family *Acipenseridae *(sturgeons), an ancient fish lineage and the sturgeon HV (AciHV1 and AciHV2) which are not sister taxa, with AciHV2 being the sister group of the ictalurid HV. Another example is provided by the eel HV (AngHV1), which grouped tightly with cyprinid HV. These authors suggested that the lack of cospeciation at deep nodes in the phylogenetic tree may indicate the existence of interspecies transmission.

There is clear evidence of interspecies transmission of OsHV-1 in marine bivalves [132,133]. Although OsHV-1 was first isolated from the moribund larval Japanese oyster *Crassostrea gigas*, a variant of OsHV-1 (termed OsHV-1var) was detected in the Manila clam *Ruditapes philipinarum *[133] and subsequently in French scallops *Pecten maximus *[132].

## 7. Aquabirnaviruses - Putative candidate for interspecies transmission but still not demonstrated

*Aquabirnavirus *is one of the four genera of the family *Birnaviridae*. The type species of the genus is infectious pancreatic necrosis virus (IPNV), the first fish virus isolated and characterised in cell culture [134]. Aquabirnaviruses have a non-enveloped, icosahedral capsid approximately 60 nm in diameter containing a bisegmented, double-stranded RNA genome. The smaller genomic segment, segment B (2.8 kb), encodes the putative RNA-dependent RNA polymerase (VP1). The larger RNA segment (segment A; 3.1 kb) contains two partially overlapping open reading frames (ORF), a large ORF encoding the polyprotein and a small ORF encoding VP5 [135].

During the 1960's most of the reports on IPNV were associated with disease in juvenile salmonids. However, in the following years, isolations of aquatic birnaviruses were made from a large number of aquatic animals, most of them from animals with no evidence of disease, reaching 80 different species, including freshwater and marine species of fish and shellfish worldwide [136]. Although no studies have been performed on the capability of aquabirnaviruses for interspecies or interclass transmission, such events would explain their wide range of host species.

Most aquabirnaviruses are antigenically related and belong to serogroup A, which includes nine serotypes (A1-A9), whereas a few isolates represent an antigenically unrelated serogroup (serogroup B) [136,137]. Six genogroups with a clear correspondence to the established serotypes have been identified [138,139]. In addition, a seventh genogroup has been proposed [140] to include yellow tail ascites virus (YTAV), isolated in Japan from an epizootic in yellowtail (*Seriola quinqueradiata*) [141]. This genogroup also includes other birnavirus strains isolated from a variety of marine fish and molluscan shellfish in Japan, which have been tentatively named marine birnavirus (MABV) [142]. The high diversity of types of this virus could be a result of a long process of adaptation to new species. In addition, recently a molecular phenomenon was discovered among aquabirnaviruses, which could **contributes **to adaptation and replication in new hosts: natural reassortment. Thus, Romero-Brey et al. [143] in an analysis of IPNV-like strains isolated from different species of wild fish captured in the Flemish Cap, Newfoundland [144], reported the existence of natural reassortant strains harbouring a WB type segment A and Ab type segment B (WB/Ab reassortant). Subsequent studies on aquatic birnaviruses isolated from wild fish in Galician coastal waters (NW Spain) [145] confirmed the presence of natural reassortants of the same type in a larger proportion of the population than in the Flemish Cap. The lack of information about segment B of most aquabirnavirus isolates reported in the literature means it is not known if reassortment is a common phenomenon in nature. Putative involvement of genetic reassortment in the spreading of aquabirnaviruses and colonisation of such a high number of aquatic species seems an interesting topic to study.

## 8. Conclusions

The wide host range shown by many viruses affecting lower vertebrates is well known. In fact, for some of them -those historically most extensively studied- the list of susceptible species is surprisingly extensive. The best example are aquabirnaviruses. However, some others are only virulent to one or to a very narrow number of species. This diversity of host specificity patterns has not been well studied, and it is therefore poorly understood at present. In this sense, the status of knowledge varies dramatically among the different viral groups. In fact, for many of them only characterisation of field isolates has been performed, focusing on natural hosts, transmission pathways, genetic variation, etc. In a few cases, however, experimental studies have been conducted to document variations in host specificity between viral species (as for betanodaviruses) or between strains within a viral species (IHNV and VHSV). For some viruses, there is field data to support interspecies and interclass transmissions (ranavirus), for others, interspecies and interclass transmission is hypothesised based on phylogenetic relationships (novirhabdovirus, herpesvirus). For some, the molecular basis of host-specific virulence and/or host specificity has been investigated (ISAV, betanodavirus).

Compared with the examples from mammalian viruses described in the introduction, there are no absolutely certain examples of host shifts in fish viruses, but there are some that have been hypothesised based on reasonable evidence. The viruses tackled in this review are the few for which some information has been documented and is available at present, and could be summarised as follows.

Ranaviruses constitute a group of viruses with a broad host range, for which the interspecies and interclass transmission has been well documented; in addition, evidence for host shifts based on phylogenies and genome analyses is also available; however, to our knowledge, the molecular determinants and/or mechanisms for host-specificity have not been investigated.

A variation in host-specificity among the four viral genotypes of betanodaviruses is well documented. In addition, molecular determinants for their host specificity patterns have been investigated using natural reassortants and chimeric recombinant viruses. From these studies, specific amino acid changes have been identified as putatively associated with differences in host specificity.

In the case of novirhabdoviruses, variations in host specificity have been demonstrated among viral strains within both species -IHNV and VHSV-, although to a higher extent within VHSV. Although not scientifically demonstrated from a molecular basis, host shift/adaptation events could be hypothesised based on phylogenetic analyses. Moreover, whole genome sequence comparisons and infectious clones of IHNV and VHSV are now available, which are being used to study, and more deeply understand, host specificity determination in these viruses.

Several studies on the molecular basis of virulence of ISA virus have indicated that changes located in the haemagglutinin (HPR) and in the fusion protein are associated with outbreaks in Atlantic salmon. Based on phylogenetic analysis, it has been hypothesised that these changes could have been involved in a change in host specificity.

Phylogenetic analysis comparing fish herpesvirus and host lineages have revealed discordances that may suggest the existence of interspecies transmission. In addition, in mollusk bivalves there is field evidence of interspecies transmission of herpesviruses.

Finally, regarding aquabirnaviruses little information other than broad host range and diversity of IPNV (and aquabirnavirus in general) genogroups, has been reported. Similarly, there is no demonstration of variations in host specificity among different viral strains, and no studies on host specificity are available; the recently demonstrated occurrence of natural reassortment among field isolates could have some implication in determining the host specificity and virulence of these viruses, and will probably be investigated in the future.

## 9. Authors' contributions

Both authors carried out the compilation and analysis of references related to the subject, as well as the writing and edition of the manuscript. All authors read and approved the final manuscript.

## 10. Competing interests

The authors declare that they have no competing interests.
